# Assessing exposure, uptake and toxicity of silver and cerium dioxide nanoparticles from contaminated environments

**DOI:** 10.1186/1476-069X-8-S1-S2

**Published:** 2009-12-21

**Authors:** Birgit K Gaiser, Teresa F Fernandes, Mark Jepson, Jamie R Lead, Charles R Tyler, Vicki Stone

**Affiliations:** 1 Edinburgh Napier University, Merchiston Campus, Edinburgh EH10 5DT, UK; 2 Cell Imaging Facility and Department of Biochemistry, University of Bristol, Bristol BS8 1TD, UK; 3 School of Geography, Earth and Environmental Science, University of Birmingham, Edgbaston, Birmingham, B15 2TT, UK; 4 Environmental and Molecular Fish Biology, The Hatherly Laboratories, University of Exeter, Prince of Wales Road, Exeter EX4 4PS, UK

## Abstract

The aim of this project was to compare cerium oxide and silver particles of different sizes for their potential for uptake by aquatic species, human exposure via ingestion of contaminated food sources and to assess their resultant toxicity. The results demonstrate the potential for uptake of nano and larger particles by fish via the gastrointestinal tract, and by human intestinal epithelial cells, therefore suggesting that ingestion is a viable route of uptake into different organism types. A consistency was also shown in the sensitivity of aquatic, fish cell and human cell models to Ag and CeO_2 _particles of different sizes; with the observed sensitivity sequence from highest to lowest as: nano-Ag > micro Ag > nano CeO_2 _= micro CeO_2_. Such consistency suggests that further studies might allow extrapolation of results between different models and species.

## Background

Nanotechnology includes the production of nanoparticles (NPs), defined as particles with three dimensions of less than 100 nm [1]. Due to their small size, NPs exhibit greater specific surface areas and surface energies, quantum related effects and generally increased surface reactivity than those of the corresponding conventional (larger) forms, leading to vastly different properties. For these reasons NPs are being increasingly employed in a variety of consumer products, including paints, cosmetics, medicines, food and suntan lotions. A number of applications also release NPs into the environment via intentional routes. For example, zerovalent iron NPs are already in use for the remediation of polluted environments [2]. Zerovalent iron NPs, however, have been shown to remove oxygen from and alter pH ground-waters, important deleterious effects resulting in unanticipated environmental impacts. It is vital that as the nanotechnology industry expands rapidly, it does so in a sustainable and ethical manner, addressing the potential impacts on human and environmental health, alongside the development of new materials and applications. This study focuses on Ag and CeO_2 _nano and micro particles. Silver NPs were developed in order to improve human health due to their anti-microbial activity for use in wound dressings and medical equipment, but they are also now being used in clothing, food processing work surfaces and even health remedies accessible via the internet. However, it is known that Ag is highly toxic to fish and other aquatic organisms [3]. CeO_2 _has been developed as a fuel additive to improve the efficiency of combustion. A number of toxicology studies suggest that CeO_2 _NP induce relatively low levels of toxicity *in vitro *[4-7]. Both silver and CeO_2 _NP are likely to be released into waste waters and the atmosphere and thus be distributed widely in the aquatic environment. The aim of this project was therefore to conduct pilot studies using CeO_2 _and silver particles of different sizes, focusing on the potential for NP to be taken up by aquatic species, human exposure via ingestion of contaminated food sources and the resultant toxic impact to the exposed organisms and cells.

## Methods

### Particles and characterisation

**Figure 1:**
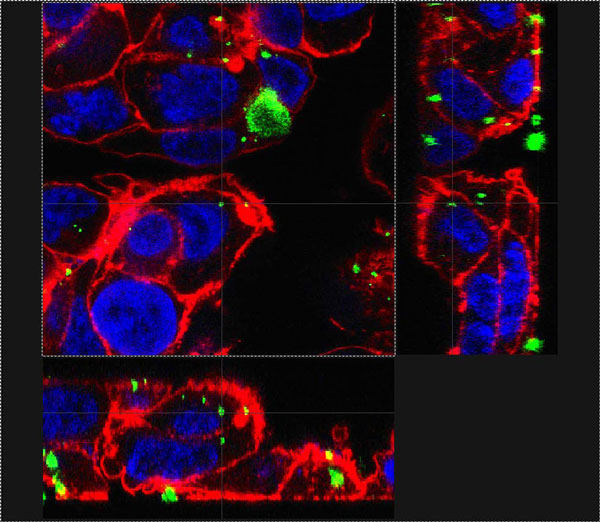
**Composite Z-stack image of C3A human hepatocyte cell line treated with silver nanoparticles**. Cells were treated for 2 h with Ag NP at a concentration of 31.25 μg/cm^2^. Red represents the F-actin cytoskeleton, blue the nuclei and green the particles. The faint grey line drawn from the particle in the center of the main frame indicates its position in the two sections on the sides and confirms its location within the cell.

Ag particles of nominal sizes 35 nm (nano Ag) and 0.6-1.6 μm (bulk Ag) diameter were purchased from Nanostructured and Amorphous Materials (USA) and dispersed without use of surfactants, capping agents or other dispersants. CeO_2 _of nominal sizes <25 nm (nano-CeO_2_) and <5 μm (bulk CeO_2_) were purchased from Sigma. These sizes were provided by the suppliers, but were investigated further by TEM, STEM (Figure 1), SEM, AFM and DLS. Other characterisation techniques included specific surface area (BET), charge (zeta potential), composition and surface chemistry (XPS, ICP-MS and UV visible spectroscopy), crystal structure (TEM and XRD) and dissolution (UF-ICP-MS). Characterisation was conducted of the pristine particles, as well as of the particles dispersed in all of the media used in the experiments described below in order to allow characteristics to be related to any observed uptake and toxicity.

### Aquatic species

*Daphnia magna *neonates were exposed for 96 h to EPA water containing 0-10 μg/ml of particles. Particles were prepared by sonicating for 30 minutes. Endpoints assessed included lethality and shedding of the carapace. Controls were treated with EPA water without the addition of particles.

Carp *(Cyprius carpio) *were kept in oxygenated, dechlorinated tap water at 10°C. For each treatment group, 8 fish were maintained in 60 l of water, 50% of which was replaced with the appropriate doses of nanoparticles every 48 h. Nano and bulk Ag particles were added to the tank water after sonication in double distilled H2O (15 minutes) at 0.01 and 0.1 μg/ml. Control fish were exposed to dechlorinated tap water without the addition of particles. After 21 d, *C. carpio *were sacrificed, and various organs were removed and processed for ICP-OES analysis to determine tissue levels of the exposed NP.

### *In vitro *human and trout cell models

The C3A human hepatocyte cell line was cultured in M2279 medium supplemented with 10% foetal calf serum (FCS), 2 mM L-glutamine, 100 Units/ml penicillin, 0.1 mg/ml streptomycin, 1 mM sodium pyruvate and 1% non-essential amino acids at 37°C and 5% CO_2_.

The Caco-2 human intestinal epithelial cell line was maintained in MEM medium supplemented with 10% FCS, 2 mM L-glutamine and 0.5 mg/ml gentamycin at 37°C and 5% CO_2_. For uptake and transport assays, cells were plated into 12-well-Transwell inserts at 500,000 cells per well and cultured for 2 weeks until a differentiated monolayer was formed.

Primary trout hepatocytes were cultured in Sigma Medium 199 supplemented with 344 mg/l NaHCO_3_, 500 mg/l CaCl_2_·2H2O, 10% FCS, 834 mg/l Hepes, 100 Units/ml penicillin and 10 μg/l streptomycin, and incubated at 12°C.

For all *in vitro *experiments, the particles were dispersed by sonication at 1 mg/ml in culture medium with additives (described above) for 15 minutes, and then diluted to the concentrations to be used in the studies.

To assess cytotoxicity, hepatocyte cells were plated at 100,000 cells per well in a 96-well-plate, incubated overnight and treated with 0-1000 μg/ml (0-625 μg/cm^2^) for 24 h. The supernatants were then analysed to assess lactate dehydrogenase (LDH) release from cells as described in [8]. Negative controls were treated with media only, and the detergent Triton X-100 was used as a positive control (100% cell death).

To assess particle uptake C3A cells plated on glass coverslips and Caco-2 cells plated on Transwell membranes were incubated with media only (negative control) or with particle suspensions at 3.125 and 31.25 μg/cm^2 ^for 2 h (C3A) or 24 h (Caco-2). Cells were stained for actin using Phalloidin-FITC and for DNA using DAPI.

## Results

### Characterisation

The sizes measured varied according the technique used as expected, but confirm the significant size difference between the bulk and nanoparticle forms. Solubility was below 1% for all samples.

### Daphnia magna

In a 96 h acute *D. magna *exposure study, nano Ag caused more mortality than bulk Ag, while CeO_2 _of both sizes did not induce any significant mortality (Table 1).

**Table 1:** Toxicity of Ag and CeO_2 _particles in *in vitro *experiments. The table lists the LC_50 _for Ag and CeO_2 _particles in *in vitro *experiments (C3A and primary trout hepatocytes), and the lowest concentration at which significant toxicity was observed in acute exposures of *D. magna *neonates. For cell cultures, doses between 3 and 1000 μg/ml were used. For the *D. magna *exposures, doses between 0.01 and 10 μg/ml were used.

	**Ag nano**	**Ag bulk**	**CeO_2 _nano**	**CeO_2 _bulk**
*D. magna *neonates (96 h)	60% mortality at 0.1 μg/ml	80% mortality at 1 μg/ml	No mortality observed	No mortality observed
C3A hepatocytes (24 h)	50 μg/ml (31.25 μg/cm^2^)	300 μg/ml (187.5 μg/cm^2^)	>1000 μg/ml (>625 μg/cm^2^)	>1000 μg/ml (>625 μg/cm^2^)
Primary trout hepatocytes (24 h)	1000 μg/ml (625 μg/cm^2^)	>1000 μg/ml (>625 μg/cm^2^)	>1000 μg/ml (>625 μg/cm^2^)	>1000 μg/ml (>625 μg/cm^2^)

### Cyprius carpio

Ag was detected in liver, intestine, gills and gall bladder after treatment with both sizes of particles. There was a trend towards higher uptake of the nano Ag than the micro sized particles. However, this was not statistically significant.

### *In vitro *cytotoxicity

Treatment with CeO_2_, at concentrations of up to 1000 μg/cm^2^, did not cause LDH release from either the C3A cell line or primary trout hepatocytes. Nano-Ag was more toxic than the bulk Ag, and primary trout hepatocytes were less susceptible to toxic effects compared with the human C3A cell line (Table 1).

### Uptake into C3A and Caco-2 cells

Both particle types at all sizes were taken up into both C3A hepatocytes (2 h) and Caco-2 intestinal epithelial cells (24 h exposure; Figure 1).

## Discussion

A number of conclusions can be drawn from this study. Firstly, the results clearly show that silver particles are more toxic than CeO_2 _particles in a variety of model species and cell types. For example, compared to CeO_2_, Ag particles caused a higher mortality in the aquatic invertebrate *D. magna *and are more cytotoxic to both trout primary hepatocytes and human hepatocyte cell lines *in vitro*. In addition to this, the silver NP were more toxic than the larger silver particles in the same aquatic invertebrate and *in vitro *cell models.

The carp studies demonstrated that the fish ingested Ag and accumulated it within the liver. These data suggested that Ag accumulation might be greater following exposure to the nano-Ag than the bulk Ag. Observations of the fish in the exposure tanks indicated that much of the uptake of the NPs into the fish may have occurred as a consequence of the fish eating agglomerated NP material, rather than uptake via the water through the gills. The uptake data suggest that either the nano-Ag could be more efficient at crossing the intestinal barrier than the bulk Ag, or that the dissolution of the nano-Ag, either in the surrounding water or the contents of the gastrointestinal tract, is greater than the bulk Ag, allowing greater uptake of free ions. Coupled with the observation that the nano-Ag is more toxic to the trout hepatocytes than the bulk Ag or the CeO_2 _particles, this suggests that the Ag NP pose a greater risk than the other particle types tested.

The Caco-2 cell model also demonstrated the potential for human intestinal epithelial cells to take up particles from the apical surface and to transport them into the cell. Both particle types and sizes were taken up, demonstrating that particles within the diet have the potential to enter the body following ingestion. Further studies of basolateral media and cell cultures are being conducted to investigate this further.

The role of physical and chemical properties of the nanoparticles in relation to their toxic impact in each model is clearly important. This has been investigated and provides the basis of future publications.

In conclusion, this study demonstrates the potential for uptake of selected metal and metal oxide NPs and larger particles via a variety of species/models, which simulate exposure via ingestion, and supports the view that ingestion is a viable route of uptake into different organism types. This study also demonstrates consistency in terms of the relative sensitivity of different models to different particles; with the observed sensitivity sequence from highest to lowest as: nano-Ag > micro Ag > nano CeO_2 _= micro CeO_2_. We propose that these models should be tested further with a wider range of particle types and animal model test systems to confirm this finding. If this holds true, it would enable a significant reduction in the requirement for toxicity testing and animal experimentation for NPs. This is important given the environmental health concerns associated with a rapidly expanding nanotechnology industry and the need to develop more comprehensive hazard data.

## Note

The peer review of this article can be found in Additional file 1.

## Competing interests

The authors declare that they do not have any competing interests.

## Authors' contributions

Birgit Gaiser conducted all of the laboratory work presented in this publication. Teresa Fernandes co-directed the Daphnia magna work and the whole project along with Vicki Stone. Mark Jepson directed the Caco-2 work. Jamie Lead directed the Characterisation. Charles Tyler directed the Carp study.  All authors contributed to the writing and editing of the manuscript.

## Supplementary Material

Additional file 1 Peer review Click here for file
