# Supplementary material for: Assessing exposure, uptake and toxicity of silver and cerium dioxide nanoparticles from contaminated environments
Source: Environ Health. 2009 Dec 21;8(Suppl 1):S2. doi: 10.1186/1476-069X-8-S1-S2 (PMC2796498; doi:10.1186/1476-069X-8-S1-S2)
Supplement: Additional file 1 — Peer review [file 1476-069X-8-S1-S2-S1.doc]

| Article ref no. | A1471-2105X SZ S2 |
| --- | --- |
| Title | **Assessing exposure, uptake and toxicity of NPs from contaminated environments.** |
| Author(s) | B Gaiser, TF Fernandes, M Jepson, J Lead, CR Tyler and V Stone |

**Ref#1**

**General comments:**

This brief manuscript succinctly summarises substantial and useful research on silver and cerium nano-particles.  I recommend publication after the authors have addressed the following points:

**Specific comments:**

Silver and cerium should appear in the title.

*Done.*

Concentration units used throughout the text are disparate and some are wrong.  All should be checked and unified to conform with Journal policy (SI?).

*Concentration units have been changed to μg/ml throughout the sections, where relevant. For cell culture, they are also expressed in μg/cm^2^, since (other than soluble substances) particles act by their presence on the cell surface, and therefore when switching between culture systems such as transwell systems and standard 96-well-plates, the equivalent dose is expressed per area of cell layer.*

Results in Table 1 indicate toxicity of Ag-nano and Ag-bulk at different concentrations and may contradict the conclusions in the text.

*Corrected – data points for mortality in* D.magna *neonates were put into wrong columns.*

More information should be provided regarding controls.

*Controls are now described in the methods section for each experiment.*

Uptake into cells (as illustrated in Fig. 1) would benefit from further explanation as to why the nano-particles are not just resting on the surface.

*This is now explained in the legend to Figure 1.*

Mention should be made of the potential for aggregation reactions, complexation, speciation and partitioning to regulate toxicological impact of, especially, the silver particles.

*This has been a major focus of this project, but the data generated is extremely complicated and is difficult to include within such a short communication. Instead, we have added a comment to the discussion to acknowledge the importance, as well as a reference to the focus of these factors in future publications.*

**Ref#2**

**General comments:**

This is an interesting paper and is recommended for acceptance following some minor revision.

**Specific comments:**

The concentrations in the text and in Table 1 appear to be inconsistent.

*This has been changed, see above.*

**Note from Editor:**

Please check that concentration units and references are in accord with the journal policy for *“Environmental Health”*

*Units have been changed, see above.*
